# Supplementary figures and images for: Multiple species animal movements: network properties, disease dynamics and the impact of targeted control actions
Source: Vet Res. 2022 Feb 22;53:14. doi: 10.1186/s13567-022-01031-2 (PMC8862288; doi:10.1186/s13567-022-01031-2)

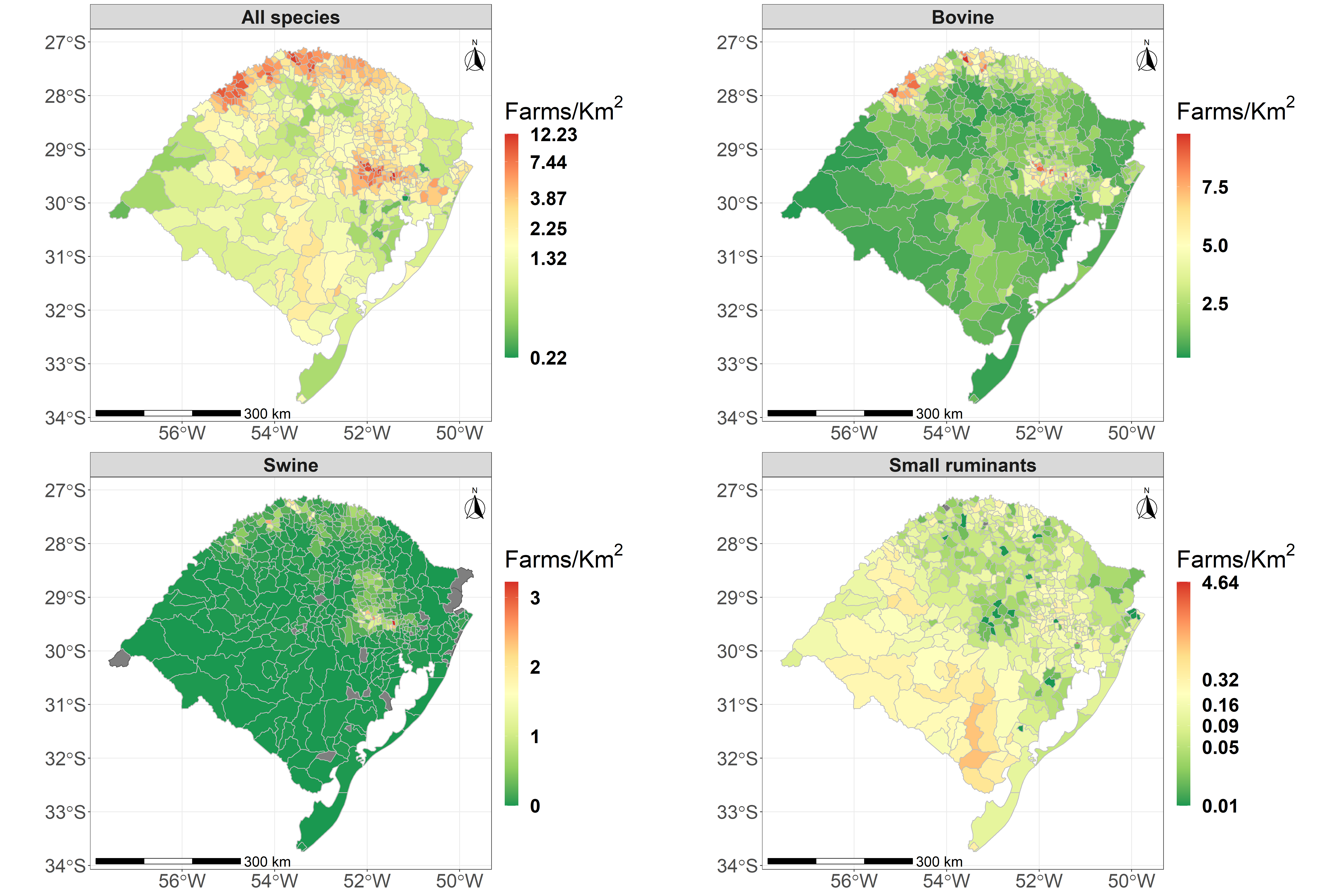

Supplement: Supplementary file 1 — Additional file 1. Spatial distribution of farms by host and municipalities by km2 in the State of Rio Grande do Sul, Brazil. [file 13567_2022_1031_MOESM1_ESM.png]

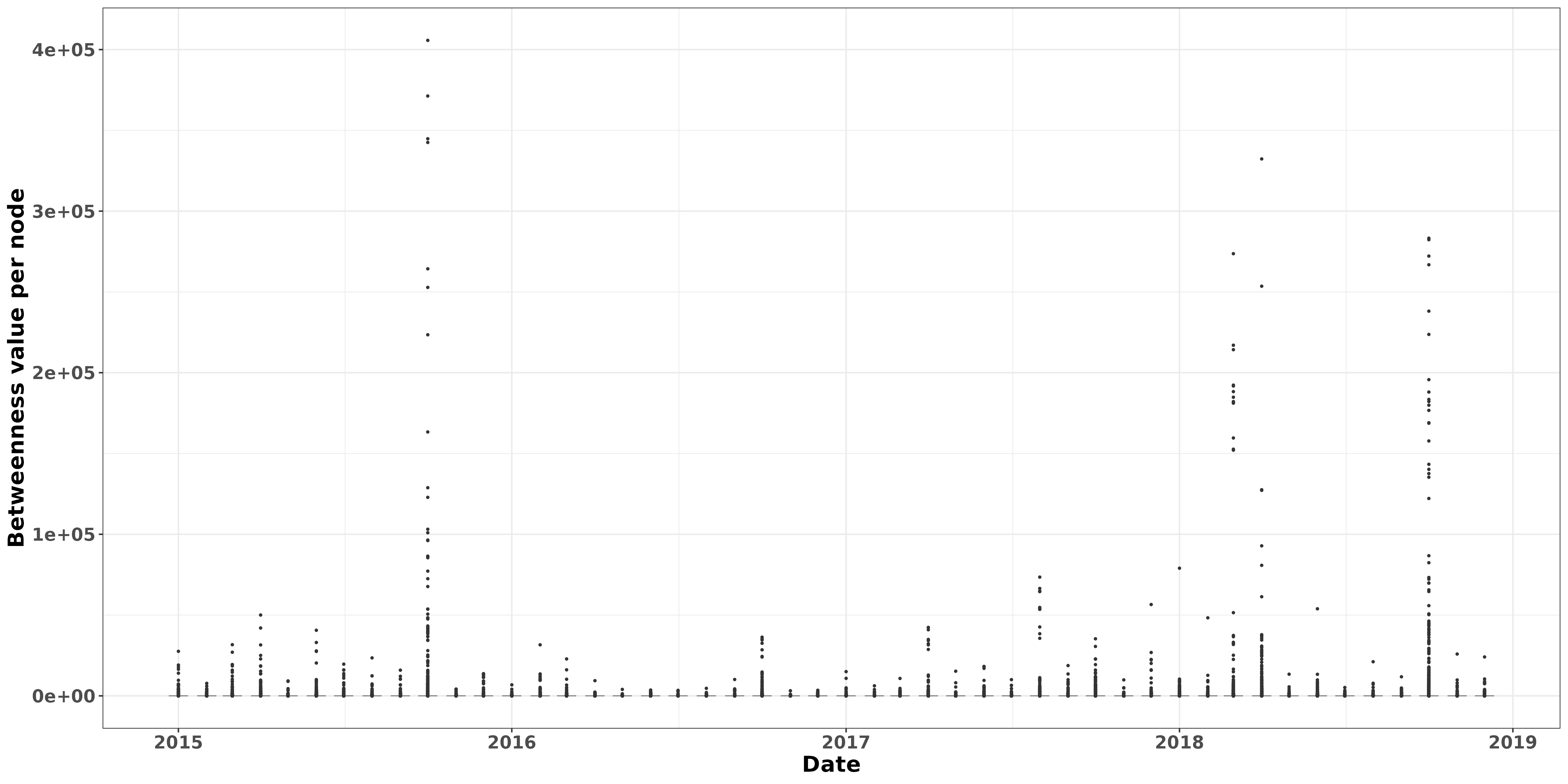

Supplement: Supplementary file 4 — Additional file 4. Monthly distribution of betweenness. [file 13567_2022_1031_MOESM4_ESM.png]

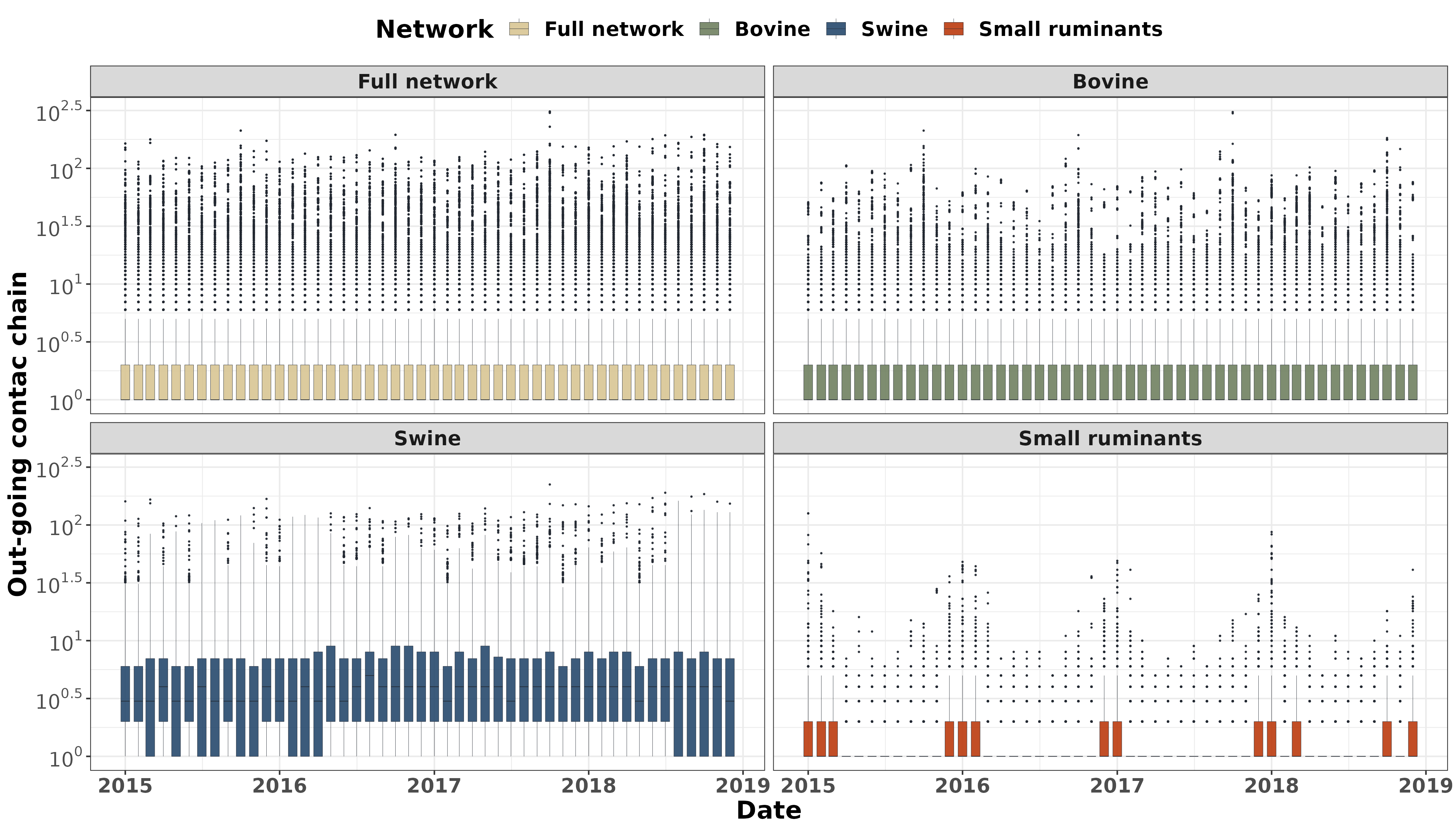

Supplement: Supplementary file 5 — Additional file 5. Monthly out-going contact chain distribution by host and species. [file 13567_2022_1031_MOESM5_ESM.png]

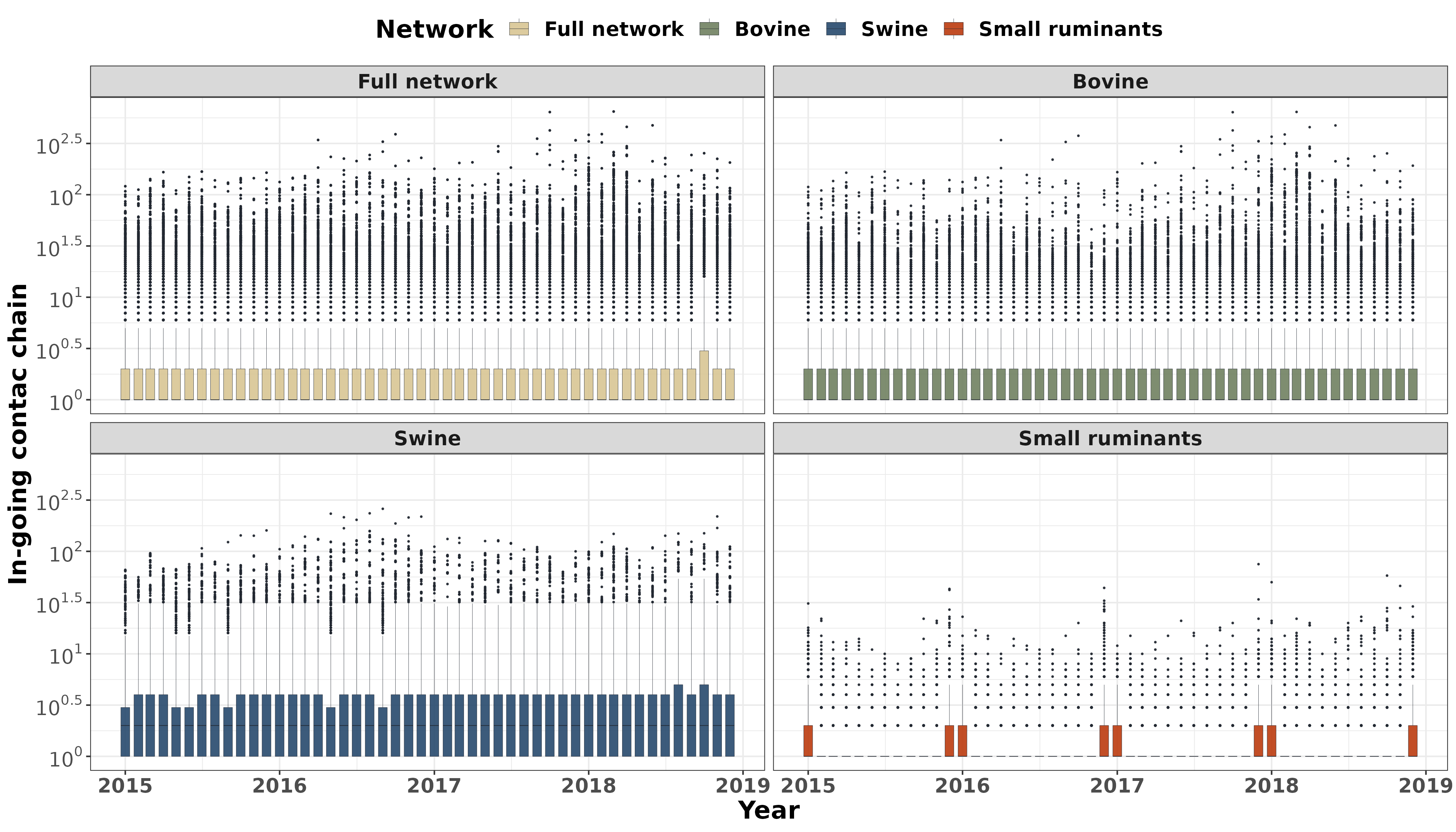

Supplement: Supplementary file 6 — Additional file 6. Monthly in-going contact chain distribution by host and species. [file 13567_2022_1031_MOESM6_ESM.png]

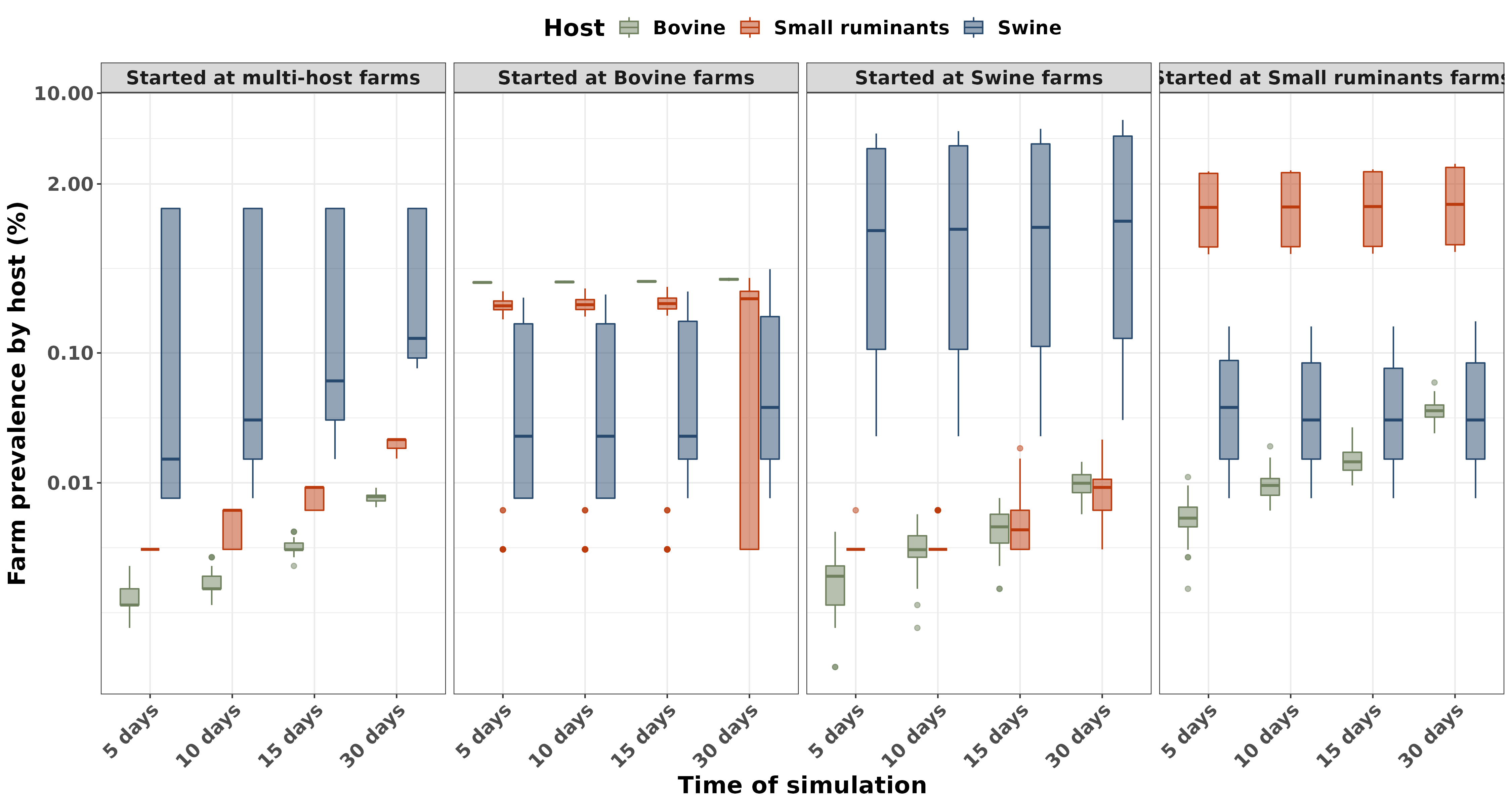

Supplement: Supplementary file 7 — Additional file 7. Number of prevalence over 30 days post-seed infection. Each title plot represents the host in which the infection was seeded. The result is represented in boxplots where the y-axis is in log10 scale and represents the number of infected farms prevalence generated through 100 simulations of each host for the first 5, 10, 15, and 30 days post-introduction. [file 13567_2022_1031_MOESM7_ESM.png]
